# Genetic Insights into Severe Obesity: A Case Study of *MC4R* Variant Identification and Clinical Implications

**DOI:** 10.3390/genes16050508

**Published:** 2025-04-28

**Authors:** Altynay Imangaliyeva, Nurgul Sikhayeva, Aidos Bolatov, Talgat Utupov, Aliya Romanova, Ilyas Akhmetollayev, Elena Zholdybayeva

**Affiliations:** 1 School of Medicine, Astana Medical University, Astana 010000, Kazakhstan; alt-er@mail.ru (A.I.); bolatovaidos@gmail.com (A.B.); 2 “National Center for Biotechnology” LLP, JSC National Holding “Qazbiopharm”, Korgalzhyn 13/1, Astana 010000, Kazakhstan; utupov_06@mail.ru (T.U.); romanovaaliya@gmail.com (A.R.); iliyas@mail.ru (I.A.); lenazhol@gmail.com (E.Z.); 3 Shenzhen University Medical School, Shenzhen University, Shenzhen 518000, China

**Keywords:** whole-exome sequencing, Kazakh family, severe obesity, mutations, BMI heritability

## Abstract

*Background/Objectives*: Severe early-onset obesity is a complex condition shaped by genetic and metabolic influences. The melanocortin 4 receptor (*MC4R*) gene plays a crucial role in energy balance, and pathogenic variants are associated with monogenic forms of obesity. This study aims to examine the clinical, metabolic, and genetic characteristics of a patient with severe early-onset obesity and his family, to assess the contribution of an *MC4R* variant to the observed phenotype. *Methods:* A 22-year-old male with severe obesity, first recognized at age 3, underwent detailed clinical, metabolic, and genetic evaluations. Laboratory assessments included insulin, lipid profile, uric acid, and IGF-1 levels. Whole-exome sequencing (WES) was performed on the patient and selected family members to identify potential pathogenic variants associated with obesity. *Results:* Clinical assessment revealed a body mass index (BMI) of 44.68 kg/m^2^, hyperinsulinemia (98.2 µIU/mL), prediabetes (HbA1c: 5.85%), dyslipidemia, hyperuricemia (421.0 µmol/L), and elevated IGF-1 levels (646.7 ng/mL). WES identified a heterozygous *MC4R*:c.216C>G (p.Asn72Lys) variant present in the patient, his mother, and maternal relatives. This variant, with a population frequency of 0.0004%, is predicted as likely pathogenic by SIFT, MutationTaster, and PrimateAI. However, its segregation pattern suggests a complex inheritance mechanism rather than classical autosomal dominant or recessive inheritance. *Conclusions*: Early genetic testing in individuals with severe obesity is essential for guiding personalized treatment strategies. Although the *MC4R*:c.216C>G variant may contribute to the patient’s metabolic profile, further functional studies are required to confirm its pathogenicity and elucidate its role in obesity pathogenesis.

## 1. Introduction

Excess body weight and obesity are major contributors to a range of health threats, such as type 2 diabetes and cardiovascular disorders, which are pressing public health issues globally [1]. The rising rates of obesity are closely linked to the increased prevalence of these chronic diseases, placing a substantial economic strain on healthcare systems due to treatment and management expenses. Additionally, obesity plays a critical role in metabolic syndrome, heightening the risk of severe health complications [2]. The financial burden extends beyond healthcare costs, as obesity also results in reduced productivity and a lower quality of life, emphasizing the urgent need for effective interventions to address its underlying causes [3]. The Body Mass Index (BMI) serves as an essential tool for assessing health risks linked to obesity, highlighting the importance of weight management in preventing adverse health outcomes [4]. Extreme obesity, classified as class 3 obesity, is characterized by a BMI exceeding 40 [5]. Data from the National Health and Nutrition Examination Survey (2017–2018) reported that 9.2% of adults and 6.1% of children were classified as severely obese [6,7,8,9]. While BMI is widely used as a diagnostic criterion, it is important to note that waist circumference is also a critical parameter, especially in the context of metabolic syndrome, where abdominal obesity serves as a major hallmark. Therefore, clinical evaluations should consider both BMI and waist circumference to more accurately assess obesity-associated risk factors, particularly those related to insulin resistance and cardiovascular disease [10].

Genetic factors play a significant role in the development of obesity, with numerous genes implicated in its onset and progression. The fat mass and obesity associated (*FTO*) gene is one of the most prominent genetic variants linked to obesity, affecting energy homeostasis and associated with various metabolic disorders [11]. Similarly, mutations in the melanocortin 4 receptor (*MC4R)* gene are linked to early-onset obesity, underscoring its role in energy balance and weight regulation. Genome-wide association studies (GWAS) have identified multiple genetic variants that increase obesity risk, shedding light on the intricate genetic framework underlying the condition. Additionally, disruptions in leptin signaling, crucial for appetite and energy balance, further exemplify the genetic underpinnings of obesity. While genetics contribute to obesity susceptibility, lifestyle choices, such as dietary habits and physical activity, also play a pivotal role. Family and twin studies have provided insights into the heritability of BMI, with estimates ranging from 20% to 90%, reflecting variations in population samples and research designs [11,12,13,14].

The *FTO* gene (officially termed FTO α-ketoglutarate-dependent dioxygenase) is a member of the AlkB-related non-heme iron and 2-oxoglutarate-dependent oxygenase superfamily. Although it shows a significant statistical link to obesity in GWAS studies, its precise physiological function is still mostly unclear [15]. Furthermore, loss-of-function mutations in FTO have been linked to serious developmental phenotypes, such as growth delays and congenital abnormalities, suggesting that its function goes beyond just adiposity [16]. Therefore, the *FTO* gene ought not to be grouped with genes that have a direct and thoroughly defined mechanistic association with monogenic obesity.

Monogenic types of obesity are usually due to uncommon variants with high penetrance, frequently resulting in severe early-onset obesity and particular syndromic characteristics. Important genes associated with monogenic obesity consist of leptin (*LEP*), leptin receptor (*LEPR*), proopiomelanocortin (*POMC*), prohormone convertase 1 (*PCSK1*), *MC4R*, single-minded homolog 1 (*SIM1*), brain-derived neurotrophic factor (*BDNF*), and its receptor *NTRK2* (commonly referred to as TrkB) [17,18]. Unlike GWAS-derived markers, these genes have recognized functions in central energy balance and appetite control, especially in the hypothalamic leptin–melanocortin pathway. For example, mutations in *MC4R* are the primary reason for monogenic obesity and are recognized to disrupt satiety signals, resulting in hyperphagia and obesity that begins early [18,19]. Consequently, a comprehensive grasp of these molecular processes is essential for providing precise diagnoses and formulating targeted therapeutic approaches.

This study utilized whole-exome sequencing (WES) to identify rare deleterious variants in genes associated with appetite suppression, adipocyte differentiation, energy homeostasis, and neuroendocrine functions. Clinical evaluations included anthropometric measurements (height, weight, BMI, and fat distribution), blood pressure, and metabolic assessments (glucose metabolism and lipid profiling). The study focused on a family with severe obesity, aiming to identify potential candidate variants. Both *de novo* and inherited variants were analyzed to explore their potential transmission to future generations, providing insight into the genetic basis of severe obesity.

## 2. Case Presentation

A 22-year-old male, born in 2002, exhibited a long-standing history of significant obesity that began in childhood. He was born at full term via a vaginal delivery. Early signs of swift weight gain were noted during infancy, with hyperphagia and significant weight increase officially documented by age three. At that stage, the proband was recommended to adopt non-pharmacological methods for weight management, such as dietary intervention and consistent exercise. As stated by his mother, the family resided in fairly good conditions, enabling her to supply the child with vitamin supplements and a diverse diet comprising fresh fruits and vegetables. At four months old, he received treatment for anemia; nonetheless, no other systemic issues were detected. By the beginning of early childhood, he consistently stayed above the 95th percentile for weight, and various dietary strategies, such as meal plans created by nutritionists and calorie limitations, produced negligible results. Starting from school age, the proband took part in general physical activities two to three times a week, though he did not join team sports like football or basketball. When he was ten years old, the family moved to a bigger city, which enhanced their socioeconomic situation and allowed for more consistent, nutritious meals. In spite of these attempts, his weight pattern deteriorated, as his BMI exceeded 35 kg/m^2^ by the age of twelve and surpassed 40 kg/m^2^ by the age of sixteen. At that point, the proband was given metformin at a dosage of 500 mg/day for three months, then 1000 mg/day for another three-month period; nonetheless, no notable weight loss occurred. Following the advice of a general practitioner and an endocrinologist, he persisted with 3-month courses of metformin at doses between 500 and 1000 mg/day, yet still without any significant clinical improvement. Upon reaching adulthood, he displayed significant obesity, marked by a BMI of 44.68 kg/m^2^. His family background showed a significant occurrence of maternal obesity, with both his mother and multiple maternal relatives exhibiting cases of early weight gain. There was no record of diabetes, metabolic syndrome, or monogenic obesity syndromes in the family history. Given the persistent and serious nature of his obesity, which continued despite various lifestyle changes and medical treatments, additional genetic and metabolic evaluations were needed.

The patient’s numerous obesity-related issues during the physical examination demonstrated the severity of his disease. Interestingly, he had acanthosis nigricans on his knees and elbows, a typical dermatological finding linked to metabolic dysfunction and insulin resistance. Furthermore, although hormonal tests ruled out adrenal illness, the development of a noticeable buffalo hump (dorsocervical fat pad), characterized by significant fat deposition in the upper back, suggested potential glucocorticoid dysregulation or Cushingoid traits. Additionally, he displayed an abdominal pannus (also known as apron belly), which is characterized by a pendulous belly and is commonly seen in cases of severe obesity and sustained weight gain. Additionally, there was edema in his lower extremities, which was probably caused by lymphatic congestion and chronic venous insufficiency. This highlighted the systemic effects of his extreme obesity. Together, these results demonstrate the intricate relationship between obesity and vascular, metabolic, and endocrine issues that calls for a multidisciplinary approach to treatment.

Numerous metabolic and hormonal abnormalities were found during laboratory testing, all performed under fasting conditions in the morning, underscoring the systemic effects of the patient’s extreme obesity. Significant hyperinsulinemia (98.2 µIU/mL) accompanied by a simultaneous fasting glucose level of 5.4 mmol/L (97.2 mg/dL) and prediabetes indicated by an HbA1c level of 5.85% suggested insulin resistance and an elevated risk of developing type 2 diabetes mellitus. Lipid abnormalities were also noted, including elevated fasting triglycerides, an increased atherogenic index, and low levels of HDL cholesterol, all of which are established risk factors for cardiovascular disease. Hyperuricemia (uric acid, 421.0 µmol/L) was also present, potentially contributing to the patient’s metabolic profile and increasing the risk of gout. Conversely, thyroid and adrenal functions appeared normal, as evidenced by thyroid-stimulating hormone (TSH, 1.19 µIU/mL) and morning cortisol (407.0 nmol/L) levels, thereby excluding primary thyroid or adrenal dysfunction as underlying causes of the obesity. However, an elevated insulin-like growth factor 1 (IGF-1, 646.7 ng/mL) level raised the possibility of disturbances in the growth hormone axis, warranting further endocrinological evaluation.

A comprehensive endocrinological evaluation was performed to assess the functional condition of the hypothalamic–pituitary axes and to explore potential hormonal factors related to the patient’s significant obesity (Table 1). The findings verified that endocrine regulation remained intact. After clonidine stimulation, growth hormone (GH) levels stayed within the normal range (14.73 ± 0.40 ng/mL before stimulation and 14.31 ± 0.17 ng/mL following stimulation; *p* = 0.003), ruling out GH deficiency. Insulin-like growth factor 1 (IGF-1) levels were persistently increased (645.48 ± 1.17 ng/mL versus 635.40 ± 1.28 ng/mL; *p* = 0.011), a result frequently seen in people with significant obesity and preserved GH responsiveness. Thyroid function was normal, indicated by levels of thyroid-stimulating hormone (TSH) and free T4. The levels of cortisol and adrenocorticotropic hormone (ACTH) taken in the morning, alongside the findings from a dexamethasone suppression test, suggested normal adrenal function and proper glucocorticoid feedback. This ruled out hypercortisolism. The hormones of the gonadal axis were also within normal physiological ranges. Total testosterone measured 15.63 ± 0.24 nmol/L in comparison to 15.49 ± 0.10 nmol/L (*p* = 0.001), while prolactin levels were recorded at 11.57 ± 0.12 ng/mL against 10.67 ± 0.06 ng/mL (*p* = 0.001). Significantly increased levels of leptin (68.5 ± 0.35 ng/mL versus 66.73 ± 0.42 ng/mL; *p* = 0.012) and insulin (98.03 ± 0.54 µIU/mL versus 95.51 ± 0.47 µIU/mL; *p* = 0.008) suggested a strong resistance to these hormones, typical of monogenic obesity types. These results were additionally backed by pituitary magnetic resonance imaging (MRI) conducted in 2022 using a 3 Tesla scanner. The pituitary gland was measured at 11 mm in the sagittal dimension, 6 mm in height, and 15 mm in the transverse dimension. The adenohypophysis and neurohypophysis were distinctly separated, and the gland exhibited a uniform structure. No unusual contrast enhancement was noted, the pituitary stalk was positioned in the midline, and no abnormal signals were found in the chiasmal-sellar area. These findings validated the lack of structural abnormalities in the pituitary gland and reinforced the idea that the patient’s obesity was not influenced by hormonal imbalance or anatomical issues within the pituitary, thereby bolstering the evidence for a monogenic basis linked to the *MC4R*:c.216C>G variant.

This case highlights the importance of early intervention and a multidisciplinary approach in managing severe obesity. The identification of the *MC4R* variant underscores the role of genetic screening in early detection and personalized treatment strategies for at-risk individuals. Further research on the functional impact of the *MC4R*:c.216C>G variant and its interaction with other genetic and environmental factors is essential.

## 3. Materials and Methods

### 3.1. Subjects

This study analyzed a family trio (father, mother, and child) with the proband suffering from severe obesity. Families were recruited based on two criteria: probands with a BMI ≥ 30 and a diagnosis of obesity before age 5. Families were excluded if any member had concomitant diseases, oncological conditions, or severe somatic pathologies, including liver and kidney failure. Data on obesity onset, chronic diseases, eating habits, and physical activity were collected using a questionnaire.

Genomic DNA from whole blood was extracted at Technopark Biogen LLP (Astana, Kazakhstan). Whole-exome sequencing was performed on a NovaSeq 6000 platform (Illumina, San Diego, CA, USA).

The study specifically aimed to identify pathogenic and likely pathogenic variants in genes previously linked to monogenic forms of obesity. Targeted genes were chosen from curated panels derived from recent research, including publications by Mohammed et al. [20] and Vourdoumpa et al. [21]. Variant annotation and interpretation were conducted according to the ACMG/AMP guidelines, retaining only those variants categorized as pathogenic, likely pathogenic, or of uncertain significance with robust computational evidence for further analysis.

One variant, for instance, was deemed likely pathogenic according to the ACMG criteria PM1, PM2, PP2, and PP4. In silico analyses were performed using the following tools: AlphaMissense (available at https://alphamissense.deepmind.com/ (accessed on 20 March 2024)), MUT Assessor (MutationAssessor; http://mutationassessor.org/ (accessed on 22 March 2024)), SIFT (Sorting Intolerant From Tolerant; https://sift.bii.a-star.edu.sg/ (accessed on 24 March 2024)), MutationTaster (http://www.mutationtaster.org/ (accessed on 26 March 2024)), DANN (Deleterious Annotation of Genetic Variants using Neural Networks; https://cbcl.ics.uci.edu/public_data/DANN/ (accessed on 28 March 2024)), PrimateAI (PrimateAI; https://platform.broadinstitute.org/primateai/ (accessed on 30 March 2024)), GenoCanyon (http://genocanyon.med.yale.edu/ (accessed on 2 April 2024)).

These collective results underscore the potential disease-causing nature of the identified variants and their significance in monogenic obesity forms.

### 3.2. Bioinformatics Analysis

The analysis workflow included BWA version 0.7.17 (Broad Institute, Cambridge, MA, USA) to align reads against the reference human genome hg38 (original GRCh38 from NCBI, December 2013); Picard version 2.18.2 (Broad Institute, Cambridge, MA, USA) to remove PCR duplicates; GATK version 4.0.5.1 (Broad Institute, Cambridge, MA, USA) for genotype calling and variant recalibration; and SnpEff version 4.3t (available at http://snpeff.sourceforge.net/ (accessed on 25 March 2024)) for variant annotation.

The following databases were used in this study: dbSNP build 151, 1000 Genomes Project Phase 3, Exome Sequencing Project (ESP6500SI-V2), dbNSFP version 3.5c, ClinVar (July 2018 version), Genome Aggregation Database (gnomAD; version 2.1.1), and Exome Aggregation Consortium (ExAC; version 0.3). Official and associated gene names were obtained from the HUGO Gene Nomenclature Committee (HGNC) database [22].

Raw variants were then filtered by region (3′ UTR, 5′ UTR, upstream, downstream, intron, intergenic, non-coding), variant type (synonymous), and minor allele frequency (1000G frequency > 0.01).

WES identified a heterozygous *MC4R*:c.216C>G (p.Asn72Lys) variant in the patient, his mother, and several maternal relatives. The *MC4R* gene, critical for energy balance and weight regulation, is linked to early-onset obesity. This variant, with a population frequency of 0.0004%, is “likely pathogenic” according to SIFT, MutationTaster, and PrimateAI predictions. The inheritance pattern in this family suggests a complex genetic contribution to the patient’s obesity, warranting further investigation.

Although WES provides comprehensive coverage of coding regions, it has limitations in detecting certain classes of genetic variation, particularly large structural variants and copy number variations (CNVs). In this study, CNVs were not reliably assessed due to the known technical constraints of WES in detecting such variants with high sensitivity and specificity. Therefore, pathogenic CNVs cannot be completely ruled out as contributors to the proband’s phenotype.

### 3.3. Variant Interpretation/Infomation

The filtered and identified genetic variants were described in accordance with the nomenclature guidelines of the Human Genome Variation Society (HGVS, http://www.hgvs.org/mutnomen (accessed on 21 March 2024)). Variant interpretation followed the five-level classification system recommended by the American College of Medical Genetics and Genomics (ACMG) and the Association for Molecular Pathology (AMP).

To confirm the validity of the identified candidate variants, Sanger sequencing was performed in the proband and their parents. Primers were synthesized for this purpose, with the following sequences: GCACAGCAATGCCAGTGA (*MC4R*-F) and AGGAGCTACAGATCACCGAG (*MC4R*-R). This comprehensive approach ensured the accurate identification, validation, and characterization of genetic variants contributing to the proband’s condition.

To further illustrate genotype–phenotype correlations, clinical information on family members carrying the *MC4R* variant (c.216C>G, p.Asn72Lys) was compiled in a comparison chart, highlighting shared and divergent phenotypic traits such as BMI, age of onset, comorbidities, and metabolic parameters. This helped assess the degree of phenotypic variability and penetrance associated with the variant within the family.

### 3.4. Ethical Compliance

All participants were fully informed about the objectives, procedures, and potential implications of the study, and their voluntary participation was secured through the process of obtaining informed consent. For children involved in the study, informed consent was provided by their parents or legal guardians. The informed consent form and procedures were reviewed and approved by the Local Ethics Committee of the National Center for Biotechnology, under Protocol #3, dated 8 July 2020. The study adhered to the ethical principles of the Declaration of Helsinki and the legal standards of Kazakhstan, ensuring academic freedom, intellectual integrity, and the right to publish findings.

## 4. Results

**Figure 1:**
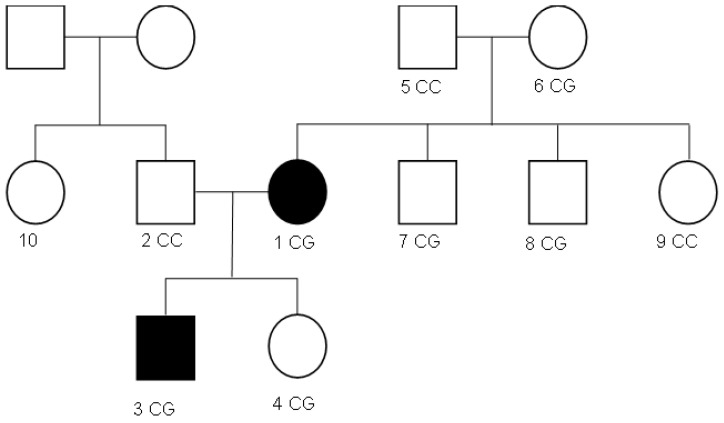
Family tree of study participants and genotypes for the *MC4R*:c.216C>G variant. The patient and his mother suffer from severe obesity, colored black and numbered 3 and 1, respectively. Trio #1 was made up of participants 5, 6 and 1. Trio #2 was made up of participants 2, 1 and 3.

This study aimed to identify severe obesity and associated metabolic disturbances. The patient, born in 2002 at 40–41 weeks gestation, was diagnosed with paratrophy at age 3 and treated for anemia at 4 months. Currently, he has a BMI of 44.68 kg/m^2^ (Table 2) and mild anemia (118 g/L hemoglobin). Clinical signs include hyperpigmentation, “buffalo hump”, “apron” sign, and lower limb edema. Lab results show prediabetes (HbA1c 5.85%), hyperuricemia, dyslipidemia, and elevated IGF-1 (646.7 ng/mL). Family analysis of 10 members spanning three generations (Figure 1) revealed a history of thrombophilia in the maternal grandmother since 2020.

Laboratory results showed normal levels of thyroid-stimulating hormone (TSH) at 1.19 µIU/mL and cortisol at 407.0 nmol/L. However, insulin levels were markedly elevated, at 98.2 µIU/mL—approximately four times the normal level. HbA1c was 5.85%, consistent with prediabetes as per the latest 2023 guidelines from the American Association of Clinical Endocrinology (AACE). Renal and hepatic function tests were within normal limits: ALT 25.6 U/L, AST 18.1 U/L, GGT 42.0 U/L, creatinine 68.0 µmol/L, urea 3.6 mmol/L, and total protein 73.2 g/L. However, the patient exhibited hyperuricemia (421.0 µmol/L) and dyslipidemia, characterized by reduced HDL cholesterol (0.7 mmol/L), hypertriglyceridemia (4.33 mmol/L), and an elevated atherogenic index (5.1, considering total cholesterol of 4.27 mmol/L and LDL cholesterol of 4.33 mmol/L). Additionally, the patient showed elevated levels of IGF-1 at 646.7 ng/mL, likely driven by hyperinsulinemia, which stimulates increased IGF-1 production. The maternal grandmother of the patient had been examined by a hematologist since 2020 for thrombophilia of an unknown origin. The study included ten family members spanning three generations (Figure 1).

The patient’s mother also has severe obesity (BMI, 43.4 kg/m^2^). According to the patient’s grandmother, the mother has been overweight since birth. The patient’s father and younger sister are healthy with a BMI 26.3 kg/m^2^ and BMI 15.01 kg/m^2^, respectively (Table 3).

Whole-exome sequencing data were obtained from nine participants, excluding participant #10. The average read count was 67,565,428, with 75.58% on-target and 96.16% target coverage (≥20×). A total of 100,893 SNPs were identified, including 12,477 synonymous, 12,218 missense, 127 stop-gained, and 26 stop-lost variants. Frameshift (293) and in-frame indels (425) were also detected. The dbSNP151 database contained 98.83% of SNPs. Using the Franklin platform (by Genoox, Genoox, Palo Alto, CA, USA), the c.216C>G (p.Asn72Lys) variant in *MC4R* was identified and validated via Sanger sequencing (Table 4). Segregation analysis revealed the variant’s presence in family members, but obesity was only observed in the proband and his mother. Further, according to multiple prediction algorithms—SIFT (0), MutationAssessor (3.86), MutationTaster (1), DANN (1), and PrimateAI (0.82)—the *MC4R*:c.216C>G variant is considered to be rare, with a population frequency of just 0.0004% (TOPMed Bravo). Additionally, the variant has a high genotype–phenotype score (0.67), suggesting that it is probably either likely pathogenic or pathogenic, according to the ACMG guidelines.

Therefore, while the relationship between the *MC4R*:c.216C>G variant and obesity in this case remains controversial, further functional and population studies are required to clarify its role.

Overall, the sequencing data suggests that the variant displays an autosomal dominant inheritance pattern, since several individuals in the maternal lineage (mother, proband, maternal grandmother, maternal uncles, and maternal aunt) show a heterozygous CG genotype, whereas the father and maternal grandfather are homozygous CC (wild-type). The proband and his sister each received the heterozygous CG variant, validating inheritance from the maternal lineage.

**Figure 2:**
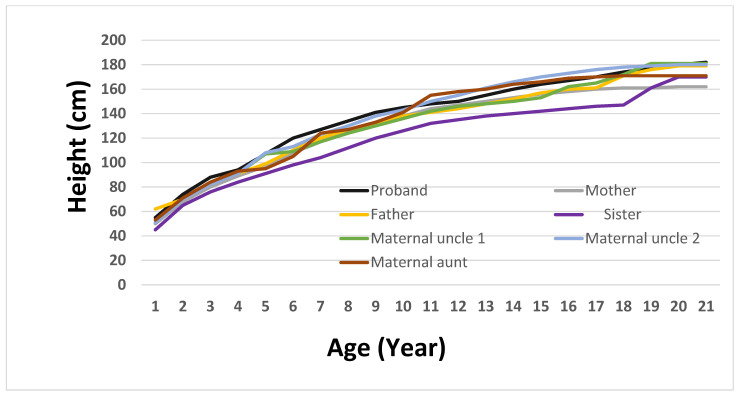
Height growth trajectories of the proband and family members up to 20 years of age.

To more accurately depict the clinical evolution of growth patterns in the family, a longitudinal height growth curve was created for the proband and nine relatives (Figure 2). This graphical illustration offers essential insight into the growth stages of children, facilitating a comparison of developmental paths. The proband exhibited early and continuous linear growth acceleration, achieving an adult height of 182 cm by age 20, which was marginally above the family average. In comparison, the sister’s curve stayed consistently below the others, whereas the mother’s and maternal uncles’ patterns exhibited standard growth trends within the familial range. Regrettably, information regarding the maternal grandparents could not be obtained, since archival medical records were lost after the breakup of the Soviet Union. By incorporating longitudinal anthropometric data, this figure visually reinforces the phenotypic differentiation of the proband and assists in understanding the relationship between genetic predisposition and growth dynamics.

**Figure 3:**
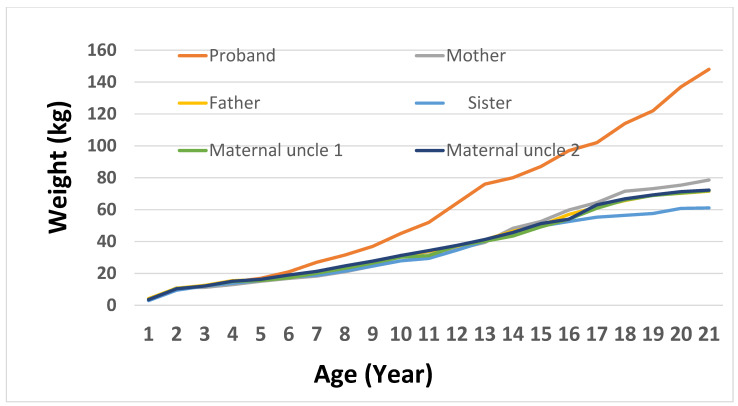
Weight growth trajectories of the proband and family members up to 20 years of age.

To evaluate growth variability in family members with the *MC4R*:c.216C>G variant, a weight trajectory analysis was performed from birth to 20 years old. The proband demonstrated the most significant and early weight increase, attaining 80 kg at 13, 114 kg at 17, and 148 kg by 20. His mother, who also possesses the variant, showed a slower increase, reaching 114 kg by the time she reached adulthood. Both maternal uncles exhibited moderate weight patterns, attaining about 85 kg by age 20, whereas the maternal grandmother displayed a gradual increase in body weight that plateaued at roughly 73 kg during her teenage years. In contrast, non-carrier family members like the father and paternal aunt kept their weight within the normal limits, reaching highs of 79 kg and 64 kg, respectively. The sister, although a carrier, showed no indications of significant weight increase, staying below 67 kg into early adulthood. Significantly, the proband’s maternal uncles and sister are all involved in physical fitness, consistently going to the gym and following organized exercise schedules. Their lifestyle decisions probably aided in sustaining healthy body weights, possibly offsetting the impacts of the *MC4R* variant. Clinical data for the maternal aunt and paternal uncle were not included, as both chose not to participate in this part of study. These findings demonstrate the diverse phenotypic manifestation of the *MC4R* variant and imply incomplete penetrance affected by supplementary genetic, environmental, or lifestyle factors (Figure 2 and Figure 3).

To evaluate the phenotypic effects of the heterozygous MC4R:c.216C>G (p.Asn72Lys) variant in the family, we analyzed clinical features of all identified carriers through whole-exome sequencing. While six family members across three generations carried the identical heterozygous MC4R variant (CG genotype), only the proband and his mother showed severe obesity (BMI > 43 kg/m2), whereas the other carriers (maternal grandmother, maternal uncles, and maternal aunt) had normal or overweight BMI readings. It should be noted that the maternal grandfather had significant cardiovascular disease history and underwent coronary artery bypass grafting at the age of 66 due to triple-vessel atherosclerotic coronary disease. He experienced chest pain at that age and was diagnosed with coronary atherosclerosis, ischemic heart disease, exertional angina (Class III), and NYHA Class II heart failure. However, due to lack of complete anthropometric data, he could not be included in the growth and weight analysis. This variation indicates incomplete penetrance and reinforces current evidence that the expressivity of MC4R mutations could be affected by genetic background, epigenetic elements, or environmental factors like diet and physical activity [23,24]. Moreover, this discovery highlights the importance of thorough segregation analysis and phenotypic profiling when interpreting variants, particularly for those with uncertain significance or probable pathogenic status [25]. An in-depth comparison of clinical characteristics among heterozygous carriers within the family (see Table 5) underscores the variability in phenotypic expression, suggesting that the MC4R variant alone may not fully account for severe obesity in all individuals and that additional genetic or environmental factors are likely involved.

## 5. Discussion

The variant *MC4R*:c.216C>G (p.Asn72Lys) was found in the proband and confirmed via Sanger sequencing in relatives. Nevertheless, its segregation pattern does not correspond with the previously outlined autosomal dominant or recessive inheritance models for *MC4R*-related obesity. In this study, the proband and his mother, as well as his maternal grandmother (individual 6) and maternal uncles (individuals 7 and 8) exhibited a heterozygous genotype for this variant. Despite this, obesity was phenotypically observed only in the proband and his mother, raising questions about variable expressivity, incomplete penetrance, or polygenic interactions influencing the phenotype.

The intrafamilial phenotypic variability seen among heterozygous carriers is consistent with previous findings that *MC4R*-associated obesity does not manifest uniformly in all individuals with a pathogenic variant. For example, Vaisse et al. reported significant clinical heterogeneity among carriers of identical *MC4R* mutations, supporting the notion of incomplete penetrance [26]. Environmental factors such as diet, physical activity, and overall energy balance have also been shown to affect phenotypic outcomes in mutation carriers [27]. In addition, the role of polygenic background, epigenetic regulation, and modifier genes may contribute to the diversity of clinical presentations in *MC4R* mutation carriers [28]. These observations underscore the importance of integrating molecular diagnostics with lifestyle and environmental assessments for a comprehensive understanding of monogenic obesity.

Previous studies have demonstrated that *MC4R*-related obesity can follow both autosomal dominant and recessive inheritance patterns. Homozygous or compound heterozygous mutations are typically associated with more severe and early-onset obesity, while heterozygous variants may exhibit a wide range of clinical expressivity [29,30]. Large cohort analyses have shown that heterozygous carriers often present with elevated BMI compared to non-carriers, although not all become obese [24,31]. While the p.Asn72Lys substitution has been previously studied in the context of the c.216C>A mutation and linked to severe obesity [29,32], no other pathogenic variants in MC4R or in other genes associated with monogenic obesity were detected in this family, strengthening the clinical relevance of the identified variant.

Although the *MC4R*:c.216C>G (p.Asn72Lys) variant was initially categorized as a variant of uncertain significance (VUS), accumulating evidence supports its reclassification as likely pathogenic. The variant results in the same amino acid substitution as c.216C>A (p.Asn72Lys), which has been functionally validated and associated with obesity in previous studies, including by Delhanty et al. [33]. Furthermore, multiple in silico prediction tools—including SIFT, MutationAssessor, MutationTaster, DANN, and PrimateAI—consistently indicate pathogenicity. Its extreme rarity in the general population, with a frequency of 0.0004% in the TOPMed database and listing as rs1015296350, is also consistent with a deleterious effect [25,29,34,35,36]. In accordance with ACMG guidelines, and based on functional equivalence, rarity, and computational support, this variant is more appropriately classified as likely pathogenic. However, the lack of direct experimental validation and large-scale genotype–phenotype correlation remains a limitation.

While the p.Asn72Lys alteration has been studied previously in relation to the c.216C>A mutation, the present study identifies a distinct nucleotide substitution, c.216C>G, which results in the same amino acid change. Despite their codon-level differences, both variants encode the identical substitution and are therefore expected to have similar effects at the protein level. Given this equivalence, no mechanistic differences in mRNA structure or splicing are currently supported by experimental evidence. Instead, attention should be directed toward the absence of functional studies that specifically assess the biological impact of the c.216C>G variant, which remains uncharacterized in clinical or laboratory settings.

It is also important to recognize the limitations of whole-exome sequencing, which does not include non-coding regulatory regions. Given that promoter variants in *MC4R* can significantly influence gene expression, it is possible that c.216C>G is in linkage disequilibrium with an undetected regulatory variant. In this scenario, the observed amino acid substitution may function as a marker for an adjacent pathogenic locus rather than being directly causal. Therefore, future studies using targeted sequencing of promoter and enhancer regions, in combination with in vitro functional assays, are essential to clarify the pathogenic mechanism of this variant.

Although computational evidence strongly suggests pathogenicity, functional studies are critical for understanding the biological effects of the p.Asn72Lys alteration on *MC4R* receptor activity. Prior research has shown that pathogenic *MC4R* variants may impair receptor trafficking, ligand binding, or downstream signaling [37,38]. The absence of functional assays remains a key limitation in interpreting the clinical impact of this variant. Planned in vitro experiments will include assessments of receptor expression, signaling activity, and ligand affinity to support formal reclassification under ACMG criteria. Furthermore, the reduced penetrance observed in heterozygous *MC4R* mutation carriers highlights the influence of additional genetic and environmental factors on disease expressivity [39,40].

*MC4R* remains the most commonly implicated gene in monogenic obesity, with heterozygous loss-of-function variants reported in 2–5% of individuals with early-onset severe obesity [41,42]. While homozygous mutations are often linked to more severe phenotypes characterized by hyperphagia and early metabolic dysregulation, heterozygous mutations can present with a broad clinical spectrum [43,44,45].

The present study also highlights key clinical markers that reinforce the monogenic nature of the proband’s condition. These include a BMI of 44.68 kg/m^2^, “buffalo hump”, prediabetes (HbA1c 5.85%), insulin levels fourfold above normal, dyslipidemia, and elevated IGF-1 (646.7 ng/mL). The patient’s mother, also a carrier, exhibited similar BMI values but with slower weight gain. In contrast, other heterozygous carriers, such as the maternal uncles and grandmother, maintained normal or moderately elevated BMI, possibly influenced by their active lifestyle habits (e.g., regular gym activity).

These findings support the role of incomplete penetrance and gene–environment interactions, including physical activity and dietary habits, in modulating phenotypic outcomes. The observation that the sister, although carrying the same variant, remains lean with a BMI of 15.24 kg/m^2^, further emphasizes the complex interplay of modifier factors.

In this study, the identified *MC4R*:c.216C>G variant was found in multiple family members with variable phenotypic expression, further supporting the concept of incomplete penetrance. Comprehensive endocrine evaluation, including pituitary axis profiling, dynamic growth hormone testing, and assessments of insulin and leptin resistance, excluded secondary causes of obesity and reinforced the likelihood of a monogenic etiology. Furthermore, the presence of thrombophilia in the maternal grandmother and cardiovascular disease in the maternal grandfather underscores the broader metabolic risk context in this family, although these comorbidities were not directly linked to the *MC4R* variant. The proband’s poor response to metformin therapy also highlights the need for genotype-informed treatment approaches, including the potential use of melanocortin receptor agonists.

## 6. Conclusions

Previously labeled as a variant of uncertain significance, increasing evidence now supports its reclassification as likely pathogenic. This encompasses its functional similarity to the c.216C>A variant, several in silico assessments suggesting a harmful impact, and its exceptional scarcity in population databases. Nonetheless, the lack of direct functional validation and extensive genotype–phenotype studies restricts conclusive interpretation.

The phenotypic differences seen among heterozygous carriers in the family highlight the necessity of including lifestyle, biochemical, and anthropometric assessments in the genetic evaluation of obesity. Consistent exercise and eating patterns probably played a role in the less severe phenotypes seen in certain carriers, such as the proband’s sister and maternal uncles. The proband’s inadequate reaction to metformin treatment and the existence of endocrine issues, like hyperinsulinemia and increased IGF-1, reinforce the necessity for genotype-informed treatment strategies.

Additionally, whole-exome sequencing did not uncover other monogenic variants but is unable to identify mutations in non-coding regulatory areas. This suggests that c.216C>G could be associated with an unnoticed pathogenic variant in the *MC4R* promoter or enhancer areas. Future research utilizing targeted sequencing and in vitro functional assays is crucial to elucidate the biological effects of this variant on receptor signaling, ligand interaction, and intracellular processing.

Ultimately, the therapeutic promise of *MC4R* agonists like setmelanotide underscores the potential of personalized medicine in treating genetically influenced obesity. This case highlights the importance of precise molecular diagnosis, thorough family segregation assessment, and meticulous phenotypic characterization to guide clinical decisions and improve long-term treatment plans.

In summary, this study’s results add to the expanding knowledge on MC4R-related obesity and highlight the need to combine clinical, genetic, and molecular information to enhance understanding of the pathogenicity and clinical effects of rare variants such as *MC4R*:c.216C>G.

## Figures and Tables

**Table 1:** Endocrine profiles before and after genetic testing.

Parameters	In 2019 (Before Genetic Testing)	In 2020 (Before Genetic Testing)	In 2021 (Before Genetic Testing)	Mean (M ± SD)	In 2022 (After Genetic Testing)	In 2023 (After Genetic Testing)	In 2024 (After Genetic Testing)	Mean (M ± SD)	*p*-Value
IGF-1 (ng/mL)	644.51	644.33	646.41	645.48 ± 1.17	639.55	635.72	635.63	635.40 ± 1.28	0.011
GH (peak after clonidine, ng/mL)	14.5	14.9	14.7	14.73 ± 0.40	14.5	14.2	14.3	14.31 ± 0.17	0.003
TSH (µIU/mL)	1.20	1.17	1.18	1.18 ± 0.13	1.16	1.15	1.18	1.16 ± 0.08	0.021
Free T4 (ng/dL)	1.11	1.11	1.12	1.11 ± 0.09	1.09	1.08	1.08	1.08 ± 0.08	0.001
Morning cortisol (nmol/L)	406.5	405.3	407.9	406.49 ± 0.47	411.5	412.4	413.1	412.27 ± 0.81	0.001
ACTH (pg/mL)	25.6	26.1	25.9	25.57 ± 0.21	26.5	26.1	26.0	26.20 ± 0.09	0.001
Dexamethasone suppression test (nmol/L)	24.2	24.3	24.7	24.38 ± 0.32	24.3	24.4	24.3	24.33 ± 0.09	0.001
Total testosterone (nmol/L)	15.7	15.5	15.8	15.63 ± 0.24	15.5	15.5	15.4	15.49 ± 0.10	0.001
Prolactin (ng/mL)	11.9	11.5	11.2	11.57 ± 0.12	11.0	10.57	10.55	10.67 ± 0.06	0.001
Leptin (ng/mL)	67.2	68.6	67.9	68.5 ± 0.35	67.12	66.85	66.78	66.73 ± 0.42	0.012
Insulin (µIU/mL)	98.1	98.4	98.6	98.03 ± 0.54	95.91	96510	95.52	95.51 ± 0.47	0.008
HbA1c (%)	5.80	5.83	5.86	5.83 ± 0.01	5.88	5.88	5.85	5.88 ± 0.05	0.001

**Table 2:** Weight gain dynamics of the patient.

Date, (DD/MM/YYYY)	Age, Years	Hight, cm	Weight, kg	BMI, kg/m^2^
16 May 2002	Date of birth	55	3.45	11.40
6 June 2002	1 month	57	4.2	12.93
16 July 2002	2 months	59	6.2	17.81
20 August 2002	3 months	63	7	17.64
17 September 2002	4 months	66.5	8	18.09
17 October 2002	5 months	67	8.6	19.16
9 January 2003	6 months	71	9.3	18.45
15 May 2003	1 year	74	10.4	18.99
16 July 2003	1 year 2 months	76	11.5	19.91
26 February 2004	2 years	80	12.5	19.53
13 September 2005	3 years	88	14.7	18.98
10 July 2006	4 years	94	16.9	19.13
11 March 2007	5 years	107	21	18.34
5 August 2008	6 years	120	27	18.75
12 July 2010	8 years	134	37	20.61
18 May 2011	9 years	141	45	22.63
16 July 2013	11 years	148	64	29.22
2 February 2015	13 years	155	80	33.30
10 August 2017	15 years	164	97	36.06
16 February 2019	17 years	170	114	39.45
10 September 2020	18 years	177	137	43.73
24 February 2022	20 years	182	148	44.68

**Table 3:** BMI indices of family members.

No.	Relationship	Date of Birth, (DD/MM/YYYY)	Hight, cm	Weight, kg	BMI, kg/m^2^
1	Mother	7 June 1981	162	114	43.44
2	Father	8 October 1978	163	70	26.35
3	Proband	16 May 2002	182	148	44.68
4	Sister	4 September 2020	81	10	15.24
5	Maternal grandfather	8 December 1957	170	83	28.72
6	Maternal grandmother	1 April 1957	164	70	26.03
7	Maternal uncle	23 September 1982	180	82	25.31
8	Maternal uncle	5 October 1986	184	85	25.11
9	Maternal aunt	25 August 1992	169	68	23.81
10	Paternal aunt	13 September 1972	160	56	21.88

**Table 4:** Sanger sequencing results.

Samples	Genotype *MC4R*:c.216 (Ref: CC)	Sequencing Data
Mother	CG	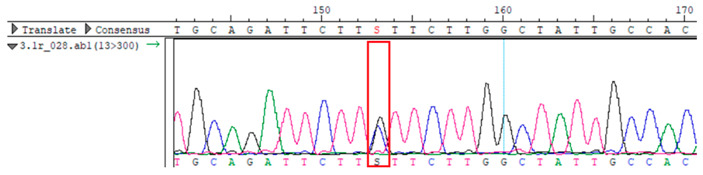
Father	CC	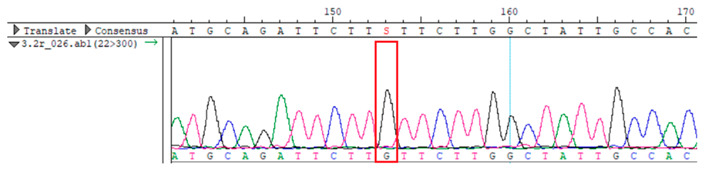
Proband	CG	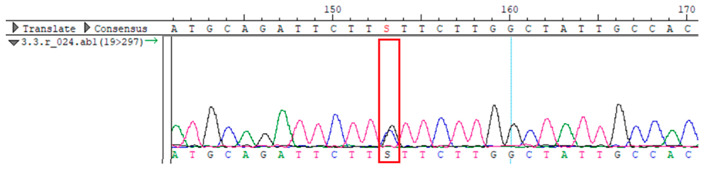
Sister	CG	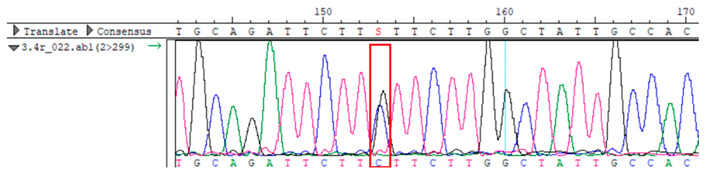
Maternal grandfather	CC	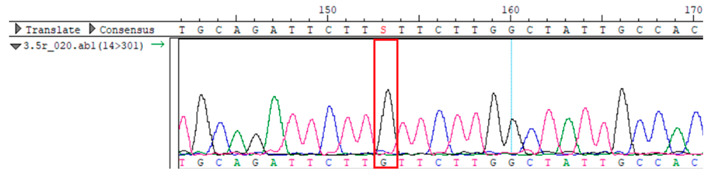
Maternal grandmother	CG	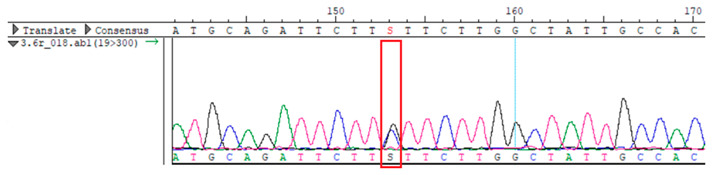
Maternal uncle	CG	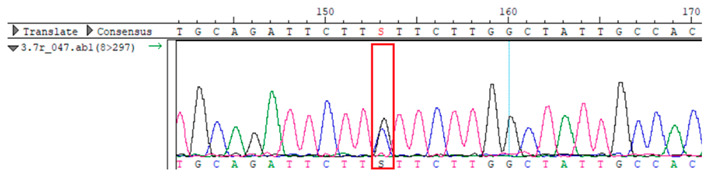
Maternal uncle	CG	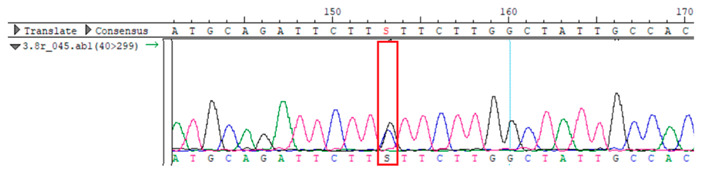
Maternal aunt	CC	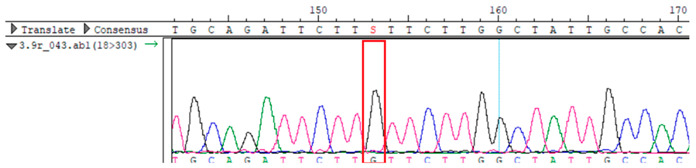

**Table 5:** Genotype–phenotype correlation and clinical features of family members carrying the *MC4R*:c.216C>G variant.

No.	Relationship	Genotype/Phenotype	Notes
1	Mother	CG/Severe obesity	Obese since childhood; BMI > 43 kg/m^2^
2	Father	CC/Normal	Maintained normal weight throughout life
3	Proband	CG/Severe obesity	Childhood-onset obesity, BMI > 43 kg/m^2^; multiple comorbidities
4	Sister	CG/Normal weight	Carrier; physically active, regular gym attendance; no signs of obesity
5	Maternal grandfather	CC/Overweight	Heterozygote status suspected but not confirmed by sequencing; underwent CABG at 66 due to triple-vessel coronary disease; diagnosed with CHD, angina (FC III), CHF (NYHA II)
6	Maternal grandmother	CG/Overweight	Carrier; diagnosed with thrombophilia since 2020
7	Maternal uncle	CG/Normal	Carrier; highly physically active, regular gym attendance; no metabolic symptoms
8	Maternal uncle	CG/Normal	Carrier; highly physically active, regular gym attendance; no metabolic symptoms
9	Maternal aunt	CG/Normal	Carrier; declined clinical examination; lifestyle unknown; presumed asymptomatic

## Data Availability

The authors confirm that the data supporting the findings of this study are available within the article.

## Abbreviations

The following abbreviations are used in this manuscript:

BMI	Body Mass Index
FTO genes	Fat mass and obesity associated genes
MC4R	Melanocortin 4 Receptor
GWAS	Genome-wide association studies
WES	Whole-exome sequencing
DNA	Deoxyribonucleic acid
SIFT	Sorting Intolerant Form Tolerant
genomAD	Genome Aggregation Database
ExAC	Exome Aggregation Consortium
UTR	Untranslated Regions
HGVS	Human Genome Variation Society
ACMG	American College of Medical Genetics and Genomics
AMP	Association for Molecular Pathology
SNFs	Single Nucleotide Polymorphisms
TSH	thyroid-stimulating hormone
AACE	American Association of Clinical Endocrinology
ALT	Alanine Aminotransferase
AST	Aspartate Aminotransferase
IGF-1	insulin-like growth factor 1
LDL	Low-Density Lipoprotein
OMIM	Online Mendelian Inheritance in Man
GENCC	Gene Curation Coalition
ORPHANET	European database dedicated to rare diseases and orphan drugs
PrimateAI	Machine-learning-based tool designed for predicting the pathogenicity of genetic variants
LLP	Limited Liability Partnership
TOPMed	Trans-Omics for Precision Medicine
HbA1c	Hemoglobin A1c (Glycated Hemoglobin)
LEP	Leptin
LEPR	Leptin Receptor
POMC	Proopiomelanocortin
PCSK1	Prohormone Convertase 1
SIM1	Single-Minded Homolog 1
BDNF	Brain-Derived Neurotrophic Factor
NTRK2 (TrkB)	Neurotrophic Tyrosine Kinase Receptor Type 2 (TrkB)
MRI	Magnetic Resonance Imaging
CNVs	copy number variations
